# Some Aspects of Enteroptosis1Read before the Toronto Medical Society, December 14, 1896.

**Published:** 1897-07

**Authors:** Charles G. Stockton

**Affiliations:** Professor of the theory and practice of medicine, and clinical medicine, Medical Department of the University of Buffalo


### SOME ASPECTS OF ENTEROPTOSIS.[1]

By CHARLES G. STOCKTON, M. D.

Professor of the theory and practice of medicine, and clinical medicine, Medical Department of the University of Buffalo.

IN THE matter of the mobility of the viscera the resources of nature have been tried. In some parts considerable motion is demanded; at the same time relative fixation is necessary. It is interesting to examine the great cavities of the body and to observe how cunningly the structures are adapted to the required motion and rest. First, the cavity of the cranium: it is doubtless needful that the brain be kept still, although from the oddities of the cerebral activities in some individuals it might be surmised that the convolutions of the brain were in a state of hopeless confusion. We are struck by the marvelous adaptation of the skull into planes, buttresses, and recesses for the safe lodgement of the delicate nervous structure. Second, the cavity of the thorax: it would seem as though nature had here reached her highest success, allowing motion to the viscera, expansion and contraction of the protecting walls while providing relative fixation for the great vital organs and encasing them in a frame-work at once unbending and elastic. Third, the abdominal cavity, with which this paper has particularly to do: motion was demanded, and in the interest of motion, strength, solidity and fixation have been largely sacrificed.

The abdominal parieties must be susceptible of great relaxation to admit of the encroachment of the diaphragm in respiration, the occasional distention of the stomach and intestines with gases, and the accommodation of the gravid uterus in the female. The underlying pelvic support avails but little when the abdominal walls are in a state of over-relaxation. It so happens that in a proportion of cases as a result of improper development, frequent gestation and artificial support, the walls of the abdomen lose tone, strength and natural contractility to such an extent that the viscera receive comparatively little external support, and the normal relations of the organ are sustained by ligaments and by delicate membranes that sometimes prove insufficient.

The tendency to displacement thus brought about is greatly favored by the conventional dress adopted by the great majority of women, and as a result we find that from the period of adolescence visceral displacements become relatively more frequent and

1. Read before the Toronto Medical Society, December 14, 1896.

in middle life are more common than is generally supposed. Out of 1,310 cases examined, Glenard found in 148 enteroptosis. Meinert found in 600 women suffering from different diseases enteroptosis in 100. Although Cruveilhier, Virchow and others have pointed out the fact of visceral displacements in the abdomen, and Dietel, in 1864, described the floating kidney and its painful symptoms, we owe to Glenard, of Lyons, the honor of having made a thorough study of enteroptosis, including its etiology, its morbid anatomy and its wide range of symptoms. Since his announcement in 1885 enteroptosis has come to be acknowledged as a definite affection, and considerable study has been given to the subject, particularly in France and Germany.

Glenard believed that the disease begins with downward displacement of the transverse colon near the hepatic flexure as a result of relaxation of the attenuated colo-hepatic ligament. For this there is probably an embryonic basis, as this portion of the large gut is the last to become fixed in place, often not until after birth, as in the colonic "physiologic" constipation of infants, first described by Jacobi, and is, therefore, the first to give way under strain. Thus not only the transverse, but the ascending colon sinks away from its normal relations, and the former is found crossing the abdomen not in the usual line, but obliquely upward and to the left, where it is usually held in position by the strong gastro-colic ligament. The acute bend produced near the hepatic flexure by this prolapse of the intestine gives rise to partial occlusion of the gut, so that the transverse colon is usually found empty and contracted. Above this point are found an unusual fecal accumulation and a partial intestinal stasis. Occasionally the transverse colon makes a bend downward about the middle of its course, but as a rule it occupies the position first described, and may be felt, Glenard claims, by palpation, making the "corde-colique transverse."

The right kidney almost invariably accompanies the colon in the descent above described, and is usually in the so-called second or third stage of movable kidney. Besides this, through stretching of the mesenteries, the small intestine is displaced downward, and not infrequently the pyloric end of the stomach, and occasionally the liver and spleen.

Briefly, so much for the anatomical condition. Now as to the factors that give rise to the deformity. It should be repeated that floating kidney is usually accompanied by the displacement of some of the other viscera. Therefore, the factors that appertain to the etiology of floating kidney belong likewise to ptosis of other abdominal viscera.

Attention is called to the introductory statement that the organs of the abdomen possess relatively poor means of fixation, and that the tendency toward a loss of position is favored by the conventional dress of women. All observers unite in the importance of the latter factor. The affection appears in women five to six times as often as in men, according to Glenard. The same ratio was found by Einhorn.[1] On this point all observers practically coincide. It is rarely found before adolescence, although W. T. Stewart[2] reports an instance in an infant eight months old as proved by laparatomy. Ewald has found movable kidney in children, and believes that it must be congenital. Similar observations are made by Lindner, Drummond and Kuttner.

It will be noted that the wearing of tight apparel usually begins with adolescence. The artificial support applied not merely by corsets, but by tight-fitting garments even when corsets are excluded lead not only to the wasting of the abdominal muscles, but also to a downward pressure made worse by nearly every movement of the trunk. My colleague, Dr. Matthew D. Mann, has long insisted upon the baneful influence exerted by apparel of this kind upon the abdominal viscera. He informs me that in his laparatomies he finds in fully 50 per cent. of cases the displacement of the transverse colon below the umbilicus, and occasionally it is found in the lower part of the abdomen.

While these causal agencies are doubtless most active, it would appear that another factor of paramount importance must not be lost to sight.

The improper muscular development of the trunk is much more common in women than in men. Of course the corset interferes with the proper development, but aside from the matter of artificial support, the improper posture in standing and sitting, resulting in the bending forward of the shoulders, the backward curving of the dorsum, and the protrusion of the abdomen produce considerable mischief. It is unusual for a young woman to hold her abdomen in proper position, and observation of children will show that the habit begins before corsets are worn. The inference is that the proper position and proper muscular development should be begun in childhood, and should be particularly taught young girls during the developmental period.

Bouveret regards neurasthenia as a cause of the muscular relaxation and Charcot has also mentioned abdominal atony as the expression of a neurosis. Stiller says "that movable kidney is found especially in the irregular type of development in which slender thorax, lax musculation and scanty adipose are prominent features." In such individuals he has found the 11th and 12th ribs very movable, and on these grounds he regards movable kidney in one sense a congenital disturbance.

Virchow first pointed out that old peritonitis with adhesions may give rise to displacement of the abdominal and pelvic organs, and not a few cases of enteroptosis are at least exaggerated by this means. The removal of large abdominal tumors that have been present for a long time is frequently followed by this sagging of the stomach and intestines through atony of the ligaments and the want of abdominal support. Recently I have had a case of this kind, which terminated in death by vomiting, as a result of traction upon the duodenum.

Pick mentions that long-continued over-distention of the bowels by gas can give rise to intestinal atony and relaxation of the abdominal walls with stagnation of the contents and auto-intoxication as a result. Many observers agree that the wasting of the fatty capsule of the kidney is largely responsible for the unnatural descent of the organ, but this is a contention that would seem to require further demonstration. The fact that in nephroptosis the right kidney is the more often involved is explained by the anatomical relations of the organ. Kendal Franks[4] discusses the matter fully. It appears that the right kidney is kept in position by the pressure of the liver above and the intestines below, and the left by the spleen and the intestines, respectively. Franks holds that the dislocation of the right kidney is due to the loss of balance between the pressure of the liver above and the intestines below, and that the fat about the kidney has little to do with the matter, as fat tissue is not solid in the living state and cannot therefore afford much support to the kidneys. The loss of balance referred to is explained by the reasons hitherto given—namely, the relaxation of the abdomen, frequent parturition, the removal of abdominal tumors, etc.

Le Gendre[5] says of the etiology of floating kidney:

(1) That it nearly always occurs in women; (2) that it is exceptional in childhood; (3) it rarely appears before adolescence; (4) it occurs most often after repeated pregnancy; (5) it frequently coincides with dyspepsia, dilatation of the stomach, gastro-intestinal atony, and enlargement of the liver; (6) it is associated with flaccidity of the abdominal walls; it is somewhat influenced by the pressure of corsets.

I have observed enteroptosis in two male children under ten years of age; repeatedly in young unmarried women who have never engaged in laborious occupations, but who have rather led lives of leisure; several times in stout, obese people, and finally in a large proportion of all females who seek relief from symptoms that apparently arise from indigestion, constipation and anemia. Some of my cases have presented symptoms of transient hydronephrosis, others obstruction of the bowels, and still others have suffered from gastric crises with pain and serious vomiting, one case at least having died as a result.

Occasionally, when the right kidney is displaced, the patient suffers pain when lying upon the left side, a symptom mentioned by Osler. With many there is complaint of abdominal pain, usually to the right of the median line, often in the tract of the right ureter. In severity the pain approaches that seen in nephritic colic. It is probably sometimes the result of kinking of the ureter and temporary hydronephrosis, but, judging from the symptoms, the pain is more often of neuralgic character and closely resembles true gastralgia, save that the location of the pain is somewhat different.

Albarran,[6] at the last meeting of the French Congress of Surgeons, says:

That he has proved by operation that the painful crisis followed by polyuria is not always dependent upon kinking of the ureter and hydronephrosis, but rather upon temporary congestion of the organ with augmentation in size. This condition would explain transient albuminuria, occasionally accompanied by casts, mentioned by Sigaud as a result of nephroptosis. He would thus explain most cases of intermittent albuminuria.

Osler[7] states that:

It may be that in persons with a debilitated and bankrupt nervous system, the tension caused by the sagging of the movable kidney may be at once felt, just as persons find the first indication of physical fagging in subjective sensation of the movements of the heart, of which in health we are not cognisant."

A certain class of patients, either with or without some of the manifestations above described, are sufferers from symptoms generally attributed to neurasthenia. One case with both kidneys floating had for years suffered from intense constipation, partial food stagnation, abdominal neuralgia, chlorosis, headache, insomnia, great irritability, and inability to engage in mental or physical activity. For her relief one ovary was removed, the rectum received thorough surgical treatment, lavage and internal gastrofaradisation were practised, with careful attention to diet and drugging, the eyes refracted by an acknowledged expert, all without real improvement to the patient. Finally, nephrorrhaphy was practised, and six months afterward the woman was practically restored to health. The bowels move regularly, the diet unrestricted, the patient sleeping well, and for the first time in eight years she enjoyed the pleasures of life, and all this without treatment of any kind.

Enough has been said to show the great frequency of enteroptosis and to present to view the wide variety of its symptomatology. It is proposed at this time to draw your attention particularly to the affection as concerns two of its phases. First, its effects upon digestion; second, its effects upon the blood.

Glenard went so far as to ascribe all digestive disturbances to faulty positions of the abdominal viscera, and his disciple, Sigaud,[8] also of Lyons, reiterates and emphasises these views. German and American physicians regard this doctrine as an evident exaggeration, but it is gradually coming to be seen that in a certain class of dyspeptics the vast majority are victims of enteroptosis to a greater or less extent. This class by no means includes all so-called nervous dyspeptics. Many of these suffer as a result of reflex irritation arising outside the abdomen. But when all known causes of functional gastric disturbances have been excluded, there remains a considerable group whose symptoms cannot easily be accounted for upon any other hypothesis than enteroptosis. Such patients are often, but not always, slender, with long, narrow chests, and show marks of corset wearing. The upper part of the abdomen is hollowed out and the abdominal pulsation is usually observable. The lower part of the abdomen is prominent, and there is often the lateral extension of the abdomen when the patient is lying upon the back, a feature pointed out by Glenard. Upon palpation the gastric splashing sound is easily produced, and the transverse colon, the "corde-colique transverse" of Glenard, may be felt over the aorta, in the region of the umbilicus, six to ten centimeters either side of the median line. This ridge is believed by Boas to be the pancreas.

Lahnsen[9], Meinert and others call attention to the tenderness over the solar plexus and to points of tenderness along the spine.

Such patients are almost invariably constipated, and suffer from a variety of digestive symptoms, prominent among which are flatulency, loss of appetite, a sensation of epigastric weight or distention which is made worse by eating, nausea and occasional attacks of pain and vomiting. The liver is usually large, or at any rate the area of hepatic dulness is increased. There is a heavily-coated tongue, a disagreeable taste in the mouth, and often an offensive breath. The complexion is muddy and the skin pigmented. The other symptoms of autointoxication should be added to the above list, among which headache and divers forms of cerebral discomfort are prominent. Examination of the stomach contents reveals delayed digestion and diminished motor activity. Free hydrochloric acid is generally found present, and the combined chlorides abundant. The total acidity is usually high, partly because of the chlorides and largely because of the organic acids resulting from fermentation. The disagreeable odor of the fatty acids is quite uniformly noticed. When free hydrochloric acid is absent, or present in small quantity, lactic acid is to be found, sometimes in considerable amounts.

It will be found necessary to introduce the aspiration tube further than usual in order to empty the stomach. The instrument must be passed 24, sometimes 28, inches beyond the teeth in order to reach the most dependent portion of the stomach. Einhorn's gastrodiaphane is of great assistance in making a diagnosis. It will be seen that the luminous area produced by the electric light finds its upper limit some distance below the free border of the ribs, and the pouching antrum-pylori is often found in the median line, its lower border several inches below the umbilicus. In order to determine the position of the stomach by percussion or palpation it is usually necessary to distend the organ with gas. It will be seen that the differential diagnosis between gastroptosis and gastroectasis is important. In point of fact it is common to find both conditions at the same time extant. It has long been known that enteroptosis is a factor in the etiology of gastric dilatation. Nevertheless, the latter condition is not always present, although the error is commonly made of regarding a case as one of alvance gastroectasis, when, in fact, it is merely gastroptosis.

It will be found that in gastroptosis patients usually do badly upon farinaceous and leguminous foods, and milk is generally objectionable. A concentrated diet consisting largely of albuminoids and the drinking of large quantities of water is most satisfactory. But little is gained by the administration of drugs, save a morning saline draught as a purgative. This is sometimes remarkably inefficient; the flushing of the colon, assisted by the proper posture of the patient, is, in not a few cases, the most reliable method of emptying the bowels.

Most of the gastric symptoms in these cases are alleviated by lavage, electricity and correction of the diet. But the patients are not cured, and there is a return of the discomfort when the treatment is abandoned. Furthermore, there comes a time when the continued employment of lavage leads to loss of weight and sometimes to deterioration of gastric secretion. On the whole, it must be admitted that this class of cases proves refractory to the ordinary method of treatment. The most hopeful course is to direct the therapeutic measures toward the relief of the enteroptosis, after which in most cases real improvement is obtained.

Now as to the second question, the relation of enteroptosis to chlorosis, it should be borne in mind that the importance of the relation was first pointed out by Glenard, and as years have gone by, numerous adherents to the doctrine that visceral displacement was causative of chlorosis have appeared. On this matter Sigaud remarks: "It is not in the study of the composition of the blood, but in that of the digestive tube, the hematopoietic organ par excellence, that one arrives at the comprehension of the pathogenesis of chlorosis, and to find the regimen and hygiene capable of its prevention and cure."

While this writer does not hold that absolutely all cases of chlorosis result from displacements of the abdominal viscera, he evidently believes that such is true of a vast majority. He points out the frequency of hemicrania and albuminuria with chlorosis in these cases, and finds that these symptoms, while resisting the conventional treatment, including iron, are promptly relieved by treatment addressed to enteroptosis.

One of the most prominent supporters of this theory is Meinert,[10] who, in his articles published in 1894 and 1895, holds that chlorosis is a secondary anemia and the direct result of a neurosis depending upon the descent of the stomach and the consequent irritation of the solar plexus.

The frequency of the affection in girls is accounted for by the injurious effects of improper clothing during the developmental period. He believes that the functions of the spleen are disturbed through nervous influences, thus accounting for the blood changes without necessarily introducing the factor of autointoxication.

Metsling[11] in six cases of chlorosis, having made examination by means of inflation with carbonic acid gas and by electric illumination, found that the stomachs were normal, both as to position and size, and came to the conclusion that gastroptosis is not a necessary attendant upon chlorosis. Breiggermann, in a dissertation published during this year, found in eight cases of chlorosis an abnormal sagging of the lower border of the stomach, while the upper border remained in normal position. He found the same condition in seven other cases where patients had never laced, and concludes that the sagging of the lower border of the stomach in chlorosis is a demonstration of the atony that accompanies the depreciated state of the blood. I believe that a fair-minded observer will admit that chlorosis is almost constantly associated with enteroptosis.

While the statement is made that patients who have downward displacement of the kidney and other manifestations of enteroptosis are free from symptoms, and seem none the worse for the deformity, my own experience has been much to the contrary. It is true that in some cases there is complaint made of no special discomfort, while yet it will be found that these individuals are below the ordinary standard of health, and a critical examination will demonstrate that, as a rule, there is disturbance in general nutrition and a depreciation in the blood as to the number of red cells, but especially as to the amount of hemoglobin.

The question naturally arises, which has precedence, chlorosis or enteroptosis? On this point I am not prepared to make a positive statement, but from observation established in my wards since beginning this study, I am inclined toward the opinion that in many cases the visceral sagging is responsible for the indigestion and autointoxication that give rise to the anemia. All will remember the statement of Sir Andrew Clark to the effect that chlorosis is a result of the stercoremia consequent upon constipation, and that the rational treatment of chlorosis must include the most careful attention to the alimentary canal.

In view of the findings of Meinert and others these expressions of Clark become the more suggestive. For those who have doubts upon this matter it is recommended that they systematically examine the position of the stomach, right kidney and transverse colon in all cases of chlorosis, resorting to modern methods of examination, and the result will prove to them whether or not the enteroptosis is the prime causative factor in the production of this form of anemia; it is certainly frequently associated with it, and doubtless has a marked bearing upon the future history of the case, both as to complete recovery and relapse.

Objections will be made that the ordinary chlorotic improves promptly upon the administration of chalybeates, which would not be the case if the etiology rested upon the visceral displacements. I have no answer to make to this objection, fully realising the importance of iron in the condition. Nevertheless, it is our duty to arrive at a correct understanding of the nature of chlorosis, and in this theory of Glenard we have at least a rational explanation for those very numerous cases of chlorosis that recur, and some of which never completely recover. It is, therefore, recommended that in cases of digestive disturbance, accompanied by chlorosis in which there is found displacement of one or more of the abdominal viscera, we rest not content with changing the diet, with treating the stomach locally, and with the administration of iron, but that we resort to the measures at our command for the restoration of the organs to their proper position, so far as possible.

What can be done for the abatement of enteroptosis? The first treatment suggested was the support of the sagging abdomen by the application of bandages and properly-fitting pads, and relief from all pressure from above downward, through the removal of corsets and other tight-fitting garments. These expedients do actually result in providing comfort to a great majority of cases, although alone not competent to overcome the pain, constipation and other prominent symptoms.

Several of my patients have worn abdominal supports, carefully adjusted, held downward by means of perineal bands, and made to exercise pressure in the lower portion of the abdomen by means of elastic. It was found necessary to have the bandage made by an experienced workman, and fitted to the body perfectly. I have never known a patient voluntarily to lay aside one of these supports. I do not think that they are curative in their effects, but they certainly are palliative. It is necessary in this condition to prevent fecal accumulation, and for that reason the diet should be concentrated, and daily evacuations secured by salines in the morning, assisted by large injections, as heretofore described.

In addition to these measures there should be practised regular Swedish gymnastics, carefully directed by a physician. Of course it is not always possible to secure skilful manipulation, but where such is available it will be found of very great use, not only for temporary relief, but also for permanent benefit in restoring the organs to place and in fixing them there.

The operator, by grasping the displaced organ and moving it upward by gentle pressure and vibration, will often succeed in its replacement. The patient may be put in a posture resembling Trendelenburg's position, and then a thorough treatment by pressure, vibration and percussion should be carried out. The objects are, of course, to improve the innervation and nutrition of the viscera. Or, the abdomen may be supported from below upward, and trunk rotation, trunk flexion and extension practised with a view to strengthen the abdominal muscles and also to improve the abdominal blood supply. The treatment may be preceded by respiratory movements and other expedients calculated to elevate the thorax and to raise the plane and strengthen the muscles of the diaphragm.

Undoubtedly ordinary spinal treatment, having for its object the improvement of the general innervation, is of value, and finally the application of the general principles of Swedish movements to the remainder of the body will be of advantage in improving the general tone and health of the individual. It will readily be seen that the unintelligent and bungling masseur may accomplish little good and much harm by these measures, if applied without a proper knowledge of anatomy and physiology. It is probable that most of the harm resulting from Swedish gymnastics is owing to the lack of professional interest and professional direction of the work. How physicians can feel that it is safe to entrust their patients even to skilful operators without personal attention to the treatment it is difficult to comprehend. Some time is given to the discussion of this side of the treatment because it is thought to be most practicable in the majority of cases, and because it is known to lead to real and lasting benefit.

I would not go so far as Leopold Fellner,[12] who claims by this means alone to be able to replace and fix the floating kidney, but I am sure that in cases of nephroptosis, under treatment by Swedish gymnastics, the position of the organ is improved and the ligaments are so strengthened that there is less irritation of the kidney and fewer symptoms resulting. In some instances relief is had although the kidney can still be palpated, but apparently occupying a higher plane in the abdomen. The same may be said of the displaced stomach and colon.

There is finally to be considered relief by operative procedures. Although Treeves has produced some relief by removal of portions of the omentum, and although Wier and others have overcome food stagnation by lessening the capacity of the dilated stomach—with the exception of nephrorraphy, there have been few operations for the relief of enteroptosis. As you know, the results of this operation have been discussed pro and con for a number of years with much difference of opinion as to the permanency of the fixation and as to the general benefit that follows it. This question is one of much interest and importance, because in many instances the restoration of the kidney to its position has resulted not only in the relief of the renal symptoms, but the stomach and intestinal symptoms as well. In such cases it is probable that there is not present a very acute bend in the colon or a very sharp flexure in the duodenum. When these latter conditions are present the benefit following the nephrorraphy cannot be expected to do more than to lessen reflex irritation and to relieve the renal crises and the transient hydronephrosis. Doubtless some of the discredit that has followed nephrorraphy depends upon the fact that the stomach and intestine remain displaced, and therefore, the pain, food stagnation and autointoxication continue. What the surgery of the future may be able to accomplish in the way of restoring the fallen alimentary tract remains to be seen.

In the case already referred to there was great displacement and some enlargement of the stomach; the colon was displaced in the ascending and in the right transverse portion. The small intestine was very much depressed, and the gastro-intestinal symptoms were striking. It was a matter of no little surprise to find that when the kidneys were restored to place that all the functions improved, and the gastro-intestinal functions were satisfactorily carried on in spite of the gastroptosis and enteroptosis that continued.

Another case:

A female nurse, widow, aged 35 years, who had always been in good health, strong and rather stout, had suffered during the past year from gastric crises at intervals of a few days or a few weeks. At these times she suffered intense pain in the epigastrium, and vomited with great severity. The crises became so frequent that her life was threatened. The stomach was somewhat displaced downward and the right kidney was floating. It was found to be exquisitely sensitive to pressure. Right nephrorraphy was done May last, since which time the patient has been perfectly relieved of her trouble, has regained her usual weight and strength and is at present actively engaged as a hard working nurse.

In this case there is some doubt as to whether the symptoms were not entirely due to reflex irritation arising from the kidney. It is possible that the sagging of the stomach had little effect in producing her symptoms. As these doubts appertain to many cases, and as it cannot be settled without an operation, I venture to recommend nephrorraphy in all cases in which the symptoms are aggravated. The procedure is likely to be followed by some benefit if not by complete relief. Following the operation the wearing of the bandage before described should be continued, and such exercises as are likely to strengthen the abdominal wall, without putting undue strain upon the new-formed kidney supports, should be practised.

1. Medical News, Sept. 19, 1896.
2. Med. Record, Feb. 9, 1895.
4. Med. Press and Circular, London, Aug. 14, 1895.
5. L'Union Medicale, Dec. 28, 1893.
6. Le Bulletin Medical, Oct. 28, 1896.
7. Lectures on the diagnosis of abdominal tumors, 1894.
8. Traite des troubles fonctionnels mecaniques de l'appareil digestif. Paris, 1894.
9. Munchener Medicinische Wochenschrift, Feb. 6, 1894.
10. Zur Aetiologie der Chlorose. Baden, 1894. Also, Sammlung Klin. Vortrag., Jan., 1895.
11. Wiener Med. Presse, 1895.
12. Wiener Med. Wochenschrift, 1896.

436 FRANKLIN STREET.

